# Anti-Cancer Effects of Zotarolimus Combined with 5-Fluorouracil Treatment in HCT-116 Colorectal Cancer-Bearing BALB/c Nude Mice

**DOI:** 10.3390/molecules26154683

**Published:** 2021-08-02

**Authors:** Geng-Ruei Chang, Chan-Yen Kuo, Ming-Yang Tsai, Wei-Li Lin, Tzu-Chun Lin, Huei-Jyuan Liao, Chung-Hung Chen, Yu-Chen Wang

**Affiliations:** 1 Department of Veterinary Medicine, National Chiayi University, 580 Xinmin Road, Chiayi 600023, Taiwan; grchang@mail.ncyu.edu.tw (G.-R.C.); lin890090@gmail.com (T.-C.L.); pipi324615@gmail.com (H.-J.L.); 2 Department of Research, Taipei Tzu Chi Hospital, Buddhist Tzu Chi Medical Foundation, 289 Jianguo Road, Xindian District, New Taipei 231405, Taiwan; cykuo863135@gmail.com; 3 Department of Nursing, Cardinal Tien College of Healthcare and Management, 112 Minzu Road, Sindian District, New Taipei 231038, Taiwan; 4 Animal Industry Division, Livestock Research Institute, Council of Agriculture, Executive Yuan, 112 Muchang, Xinhua Dist, Tainan 71246, Taiwan; mytsai@mail.tlri.gov.tw; 5 Graduate Institute of Bioresources, National Pingtung University of Science and Technology, 1 Shuefu Road, Neipu, Pingtung 91201, Taiwan; 6 Bachelor Degree Program in Animal Healthcare, Hungkuang University, 6 Section, 1018 Taiwan Boulevard, Shalu District, Taichung 433304, Taiwan; ivorylily99@gmail.com; 7 General Education Center, Chaoyang University of Technology, 168 Jifeng Eastern Road, Taichung 413310, Taiwan; 8 Division of Gastroenterology, Department of Internal Medicine, Chang Bing Show Chwan Memorial Hospital, 6 Lugong Road, Lukang Township, Changhua 505029, Taiwan; 9 Division of Cardiology, Asia University Hospital, 222 Fuxin Road, Wufeng District, Taichung 413505, Taiwan; 10 Department of Medical Laboratory Science and Biotechnology, Asia University, 500 Lioufeng Road, Wufeng District, Taichung 413305, Taiwan; 11 Division of Cardiovascular Medicine, China Medical University Hospital, 2 Yude Road, North District, Taichung 404332, Taiwan; 12 College of Medicine, China Medical University, 91 Hsueh-Shih Road, North District, Taichung 404333, Taiwan

**Keywords:** 5-fluorouracil, colorectal adenocarcinoma, inflammation, metastasis, zotarolimus

## Abstract

Zotarolimus is a semi-synthetic derivative of rapamycin and an inhibitor of mammalian target of rapamycin (mTOR) signaling. Currently, zotarolimus is used to prolong the survival time of organ grafts, but it is also a novel immunosuppressive agent with potent anti-proliferative activity. Here, we examine the anti-tumor effect of zotarolimus, alone and in combination with 5-fluorouracil, on HCT-116 colorectal adenocarcinoma cells implanted in BALB/c nude mice. Compared with the control mice, mice treated with zotarolimus or zotarolimus combined with 5-FU showed retarded tumor growth; increased tumor apoptosis through the enhanced expression of cleaved caspase 3 and extracellular signal-regulated kinase (ERK) phosphorylation; reduced inflammation-related factors such as IL-1β, TNF-α, and cyclooxygenase-2 (COX-2) protein; and inhibited metastasis-related factors such as CD44, epidermal growth factor receptor (EGFR), transforming growth factor β (TGF-β), and vascular endothelial growth factor (VEGF). Notably, mice treated with a combination of zotarolimus and 5-FU showed significantly retarded tumor growth, reduced tumor size, and increased tumor inhibition compared with mice treated with 5-FU or zotarolimus alone, indicating a strong synergistic effect. This in vivo study confirms that zotarolimus or zotarolimus combined with 5-FU can be used to retard colorectal adenocarcinoma growth and inhibit tumorigenesis. Our results suggest that zotarolimus may increase the chemo-sensitization of tumor cells. Therefore, zotarolimus alone and zotarolimus combined with 5-FU may be potential anti-tumor agents in the treatment of human colon adenocarcinoma. Future research on zotarolimus may lead to the development of new therapeutic strategies.

## 1. Introduction

With one to two million new cases diagnosed each year, colorectal cancer (CRC) is the third most common cancer and the fourth most common cause of cancer-related death in the world [1]. While surgical resection is the most effective therapeutic strategy for all stages of localized CRC [2], it may not eliminate all cancerous cells. Moreover, the prognosis of patients with advanced CRC after surgery remains poor. About 50% of advanced-stage CRC patients experience tumor regrowth and recurrence [3]. Post-surgery adjuvant treatments such as chemotherapy have reduced recurrence and increased survival [4]. Chemotherapeutics including doxorubicin, fluorouracil, cisplatin, and mitomycin are generally used to kill residual CRC cells after surgery [5]. In addition, studies have indicated that there is a synergistic effect when 5-fluorouracil (5-FU) treatment is used in combination with resveratrol or rapamycin to inhibit tumor growth [6,7].

Numerous studies have examined the mammalian target of rapamycin (mTOR) signaling pathway as a target of cancer therapy [8,9]. The mTOR signaling pathway is critical for the metabolism and physiology of mammals. Deregulated mTOR is associated with many pathophysiological conditions such as aging, Alzheimer’s disease, diabetes, obesity, and cancer [10,11]. mTOR forms two complexes that are distinct in both structure and function: the mammalian target of rapamycin complex 1 (mTORC1), which regulates cell growth and metabolism, and the mammalian target of rapamycin complex 2 (mTORC2), which controls cell proliferation and survival [12]. Over-activation of mTORC1 can promote tumor formation, proliferation, and metastasis, while mTORC2 regulates the expression of mTORC1 through the mTORC2/AKT/TSC/Rehb pathway. Activating the PI3K/AKT/mTORC1 pathway also activates the downstream S6K1, which initiates a series of related cellular physiological responses including vascular and tumor cell proliferation and other pathways linked to tumor formation [13]. Therefore, it is important to develop new drugs that inhibit mTOR activation and treat tumors.

Zotarolimus is an immunosuppressant. It is a semi-synthetic derivative of rapamycin that prevents allograft rejection and is also used in drug-eluting stents (DESs) to reduce post-angioplasty restenosis [14,15]. Rapamycin, however, can produce adverse effects such as immunodeficiency and hypertension, as well as cardiac, vascular, lipid metabolic, testicular and epididymal, dermatological, obstetric and gynecological, ocular, and neurological problems [16,17]. Thus, it is safer to continually administer zotarolimus than rapamycin given the lower risks of metabolic, diabetic, and hyperglycemic adverse events [11,17,18]. Zotarolimus was initially designed to be used in stents with phosphorylcholine as a carrier [19]. To create zotarolimus from rapamycin, a tetrazole ring is used as a substitute for the native hydroxyl group at position 42 in rapamycin. Zotarolimus has limited water solubility because of its lipophilicity [20]. The low water solubility prevents the compound from being rapidly released into circulation from a coronary stent because the dissolution of the drug from the stent is partly rate limited. This slow rate of release and subsequent diffusion helps to maintain therapeutic drug levels eluting from the stent. The majority of research on the use of zotarolimus focuses on the treatment of cardiovascular diseases, with few studies addressing its potential use for other diseases [21,22]. Another mTOR inhibitor, everolimus, has been found to produce direct anti-tumor effects through cell cycle arrest and increased apoptosis as well as anti-angiogenic activity in preclinical models [23]. Everolimus also reduces the expression of vascular endothelial growth factors (VEGF) in tumor-derived ovarian carcinoma in mice and in gastric cancer cells in vitro and in vivo [24]. A study on nude mice with subcutaneous human HCT-116 colon tumor xenografts treated with everolimus observed the direct inhibition of tumor growth and a dose-dependent decline in phospho-S6 kinase levels, an mTOR target [25]. Zotarolimus, which exhibits a mechanism of action similar to that of everolimus, could be used in CRC treatment. 5-FU is widely used in CRC treatment, although its clinical use is limited owing to potential drug resistance [26]. Studies have shown that the anti-cancer efficacy of 5-FU increased with the dose [27,28], but the cytotoxic effect on normal cells induced unacceptable levels of toxicity in patients [29]. To overcome this, 5-FU should be combined with other anti-cancer drugs with different mechanistic actions [27].

Patients with CRC are mainly treated with chemotherapy. However, certain patients do not respond to this therapy or may initially respond well and gradually show signs of a relapse. Increasing drug doses triggers adverse drug reactions or the development of drug resistance [28]. Thus, it is crucial to develop new drugs or a combination of drugs for CRC treatment. Although the pyrimidine analog 5-FU is widely used to treat cancers, consequent drug resistance severely limits its clinical use in lung cancer treatment [26]. Thus, this study analyzes the anti-tumor effects of zotarolimus, an mTOR inhibitor, on HCT-116 cells and the synergistic effects of zotarolimus when combined with 5-FU. The study was conducted on an HCT-116 human CRC cell line implanted in BALB/c nude mice. To explore the therapeutic properties of zotarolimus and mTOR drugs for CRC, we examined tumor growth and various aspects related to tumor development, including apoptosis, inflammation, and metastasis, with the objective of expanding the number of drugs available for CRC treatment.

## 2. Results

### 2.1. Zotarolimus Retards Tumor Growth

**Figure 1:**
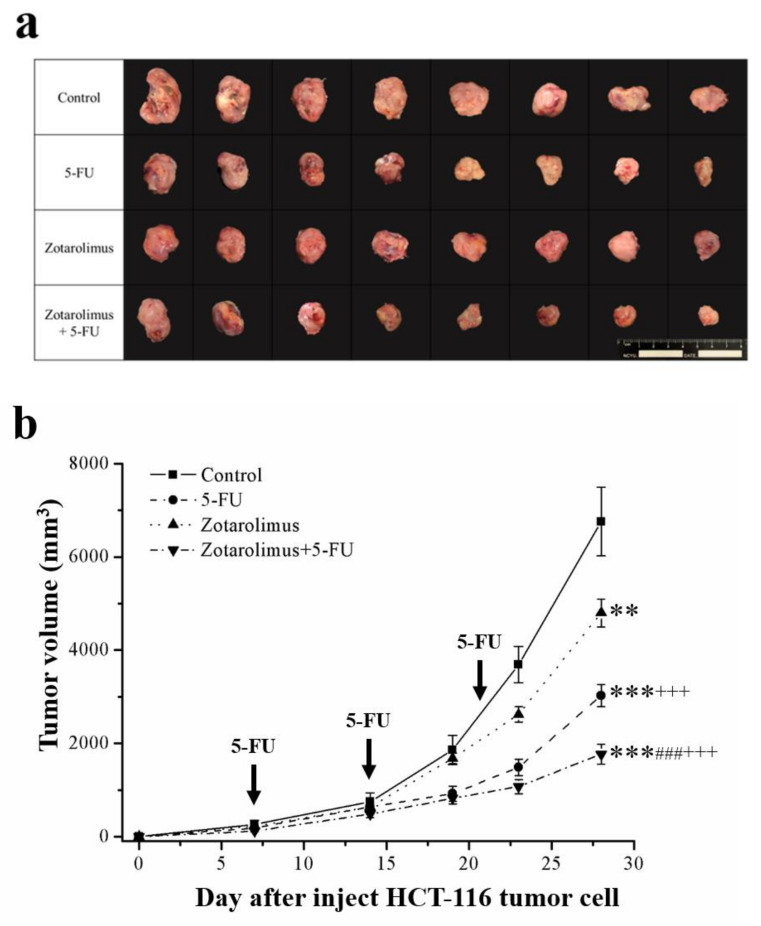
Relative volume of tumors. (**a**) Photographs of tumors excised after sacrifice on day 28. (**b**) Volumes of HCT-116 tumor masses from BALB/c nude mice administered different treatments: control (saline), 5-FU (50 mg/kg/week), zotarolimus (2 mg/kg/day), and zotarolimus (2 mg/kg/day) combined with 5-FU (50 mg/kg/week). All data are presented as mean ± standard deviation, *n* = 8 per group. ** *p* < 0.01 and *** *p* < 0.001 compared with the control group. ^###^ *p* < 0.001 compared with the 5-FU-treated group. ^+++^ *p* < 0.001 compared with the zotarolimus-treated group. All treatments were initiated on day 7 when the tumors were detected.

Previous studies suggest that 5-FU and zotarolimus have anti-cancer activity [27,30]. Accordingly, we observed reduced tumor growth and volume in the groups treated with 5-FU, zotarolimus, and a combination of zotarolimus and 5-FU compared with the control group (Figure 1a). In addition, the time course of responses revealed that the decrease in tumor growth was significantly greater in the mice treated with 5-FU (*p* < 0.001), zotarolimus (*p* < 0.01), and zotarolimus combined with 5-FU (*p* < 0.001) than in the control mice (Figure 1b). Combining zotarolimus with 5-FU led to a greater decrease in tumor growth, indicating an obvious synergistic function. Compared to the control group, the tumor inhibition rate increased by 62.1% in the 5-FU-treated group, 36.5% in the zotarolimus-treated group, and 81.5% in the group treated with a combination of zotarolimus and 5-FU (Table 1). Therefore, zotarolimus combined with 5-FU is a powerful inhibitor of the growth of human murine colorectal carcinoma.

### 2.2. Zotarolimus Increases Number of TUNEL-Positive Cells

**Figure 2:**
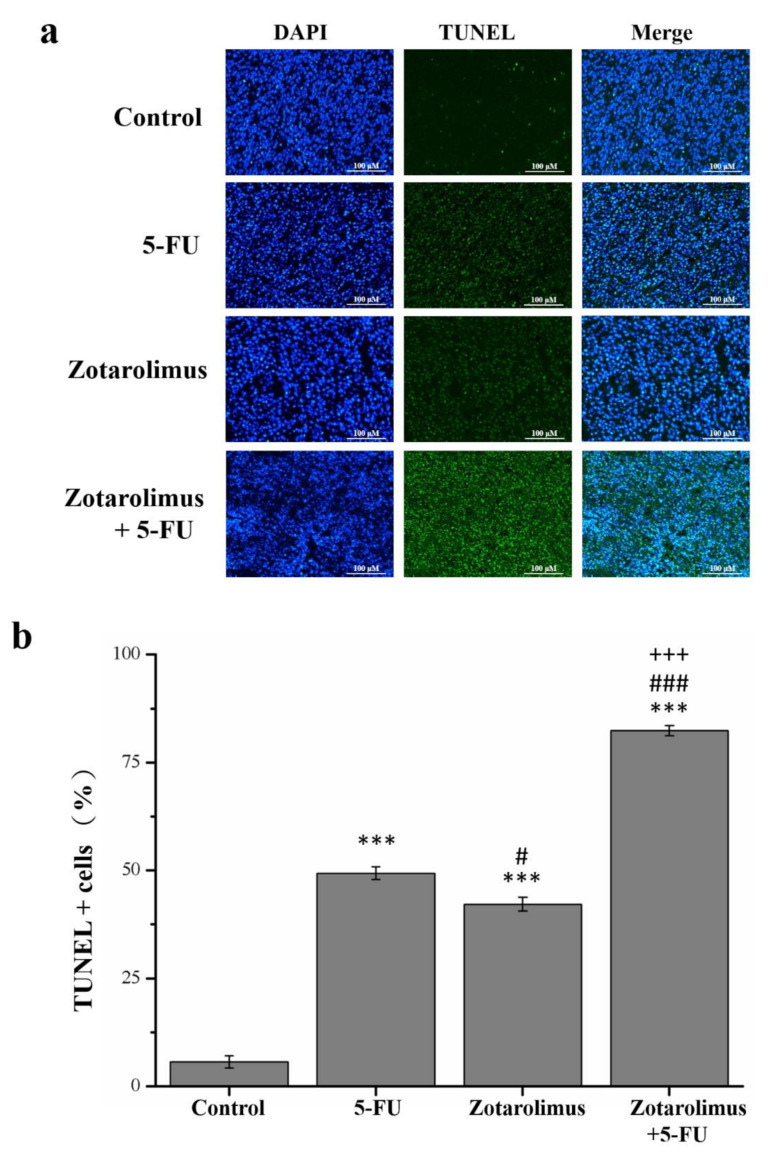
Analysis of apoptosis in HCT-116 tumors by TUNEL/DAPI staining (×200). (**a**) Images of TUNEL and DAPI staining and merge. (**b**) Percentage of TUNEL positive cells in the HCT-116 tumor mass of control BALB/c nude mice and mice administered different treatments: 5-FU (50 mg/kg/week), zotarolimus (2 mg/kg/day), and zotarolimus (2 mg/kg/day) combined with 5-FU (50 mg/kg/week). All data are presented as mean ± standard deviation, *n* = 8 per group. *** *p* < 0.001 compared with the control group. ^#^ *p* < 0.05 compared with the 5-FU-treated group. ^###^ *p* < 0.001 compared with the 5-FU-treated group. ^+++^ *p* < 0.001 compared with the zotarolimus-treated group. All treatments were initiated on day 7 when tumors were detected. Scale bars = 50 µm.

Apoptotic cells undergo extensive DNA degradation during the late stage of apoptosis, and the TUNEL assay detects such cells [31]. Our findings indicated that TUNEL positive cells increased 8.8 (*p* < 0.001), 7.5 (*p* < 0.001), and 14.7 (*p* < 0.001) times (Figure 2b) in mice treated with 5-FU, zotarolimus, and zotarolimus combined with 5-FU, respectively (Figure 2a). These values were significantly greater than those observed in the control group. The number of TUNEL-positive cells was significantly lower in the zotarolimus-treated group than in the 5-FU-treated group. Moreover, the number of TUNEL-positive cells in mice treated with zotarolimus was lower than in those treated with 5-FU (by 14.6%) and zotarolimus combined with 5-FU (by 48.8%). These results indicated that both zotarolimus and a combination of zotarolimus and 5-FU increased the degree of apoptosis, and the combination treatment had the greatest effect.

### 2.3. Zotarolimus Increases Apoptosis-Related Protein Expression

**Figure 3:**
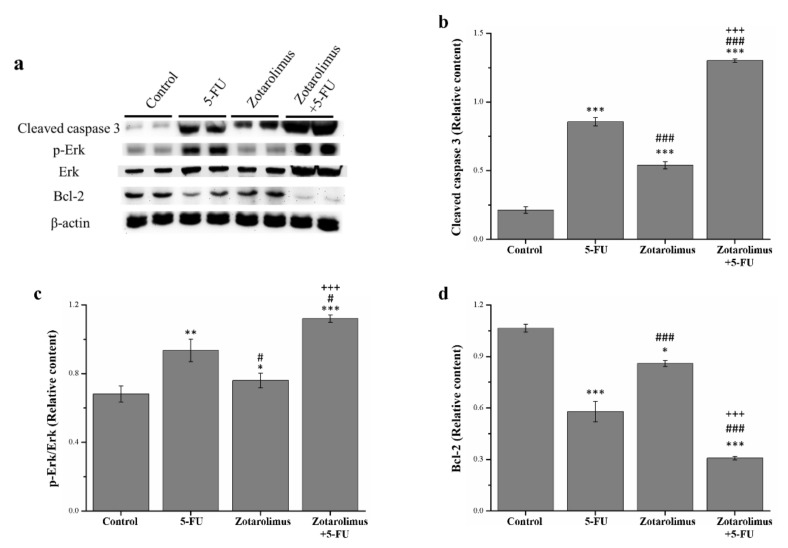
Analysis of apoptosis-related proteins including (**a**) representative Western blot showing the levels of apoptosis-related proteins extracted from the tumors; (**b**) cleaved caspase 3 expression; (**c**) ERK phosphorylation; and (**d**) Bcl-2 expression in the HCT-116 tumor masses taken from BALB/c nude control mice and mice administered different treatments: 5-FU (50 mg/kg/week), zotarolimus (2 mg/kg/day), and zotarolimus (2 mg/kg/day) combined with 5-FU (50 mg/kg/week). All data are presented as mean ± standard deviation, *n* = 8 per group. * *p* < 0.05, ** *p* < 0.01, and *** *p* < 0.001 compared with the control group. ^#^ *p* < 0.05 and ^###^ *p* < 0.001 compared with the 5-FU-treated group. ^+++^ *p* < 0.001 compared with the zotarolimus-treated group.

Apoptosis is a process of programmed cell death and is critical in the development and survival of living organisms. In this sequentially regulated suicidal program, cells activate specific enzymes that dissolute their own nuclear component and the various protein components of nucleus and cytoplasm. [32]. For the analysis, we selected apoptosis-related proteins including cleaved caspase 3, extracellular signal-regulated kinase (ERK), phosphorylated ERK, and anti-apoptosis protein B-cell lymphoma 2 (Bcl-2) (Figure 3a). The Western blotting revealed that, compared with the control group, the expression of cleaved caspase 3 and phosphorylated ERK was significantly higher in the groups treated with 5-FU (cleaved caspase 3: *p* < 0.001; phosphorylated ERK: *p* < 0.01) and zotarolimus combined with 5-FU (cleaved caspase 3: *p* < 0.001; phosphorylated ERK: *p* < 0.001) (Figure 3b,c). In addition, the zotarolimus-treated mice had a higher level of cleaved caspase 3 (*p* < 0.001) and ERK phosphorylation (*p* < 0.05) than the control mice. 5-FU- and zotarolimus-treated groups had a lower expression of cleaved caspase 3 (5-FU: *p* < 0.001; zotarolimus: *p* < 0.001) and ERK phosphorylation (5-FU: *p* < 0.05; zotarolimus: *p* < 0.001) than the group treated with zotarolimus combined with 5-FU. Mice treated with zotarolimus combined with 5-FU had the highest expression of cleaved caspase 3 and ERK phosphorylation. Bcl-2 is widely considered to be a suppressor of apoptosis. We found that, compared with the control group, Bcl-2 expression was lower in the 5-FU (*p* < 0.001), zotarolimus (*p* < 0.05), and 5-FU combined with zotarolimus groups (*p* < 0.001) (Figure 3d). However, Bcl-2 expression in the zotarolimus group was higher than that in the 5-FU-treated group (*p* < 0.001) and in the group treated with zotarolimus combined with 5-FU (*p* < 0.001), which had the lowest Bcl-2 expression. The results suggested that zotarolimus and zotarolimus combined with 5-FU enhanced the expression of apoptosis-related proteins and inhibited anti-apoptotic protein expression. Further, zotarolimus combined with 5-FU exhibited the maximum effect on apoptosis.

### 2.4. Zotarolimus Inhibits Production of Inflammation-Related Factors

**Figure 4:**
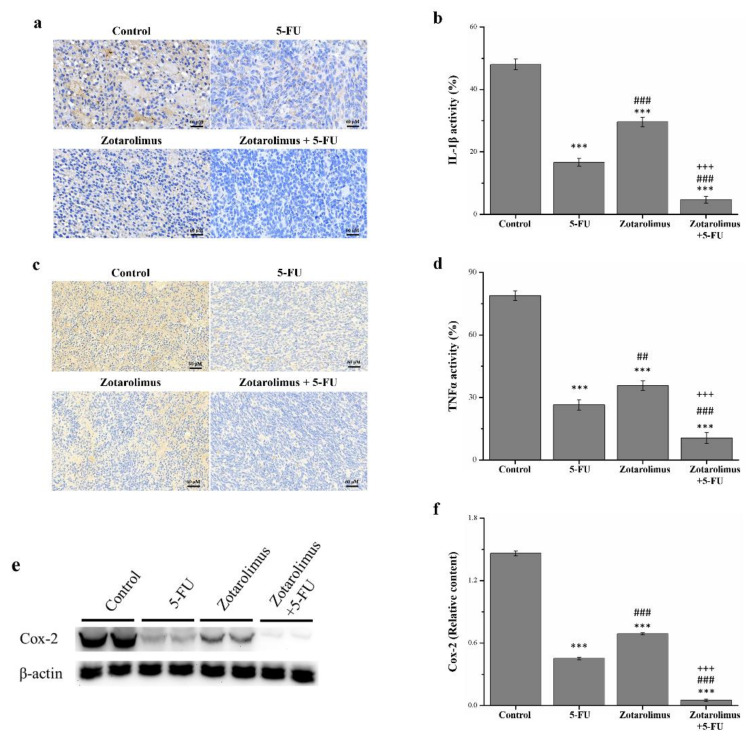
Immunohistochemical expression of (**a**) IL-1β and (**c**) TNF-α in HCT-116 tumors from BALB/c nude mice and the comparative immunohistochemical expressions of (**b**) IL-1β and (**d**) TNF-α. (**e**) Western blot analysis and (**f**) expression level of COX-2. All experiments were conducted on the HCT-116 tumor masses from BALB/c nude mice. Findings are reported for the control group and treatment groups: 5-FU (50 mg/kg/week), zotarolimus (2 mg/kg/day), and zotarolimus (2 mg/kg/day) combined with 5-FU (50 mg/kg/week). All data are presented as mean ± standard deviation, *n* = 8 per group. *** *p* < 0.001 compared with the control group. ^##^ *p* < 0.01 and ^###^ *p* < 0.001 compared with the 5-FU group. ^+++^ *p* < 0.001 compared with the zotarolimus group. Scale bars = 60 µm.

Cytokines regulate a wide range of processes in the pathogenesis of cancer. Inflammation plays a key role in the integrity and sustenance of multicellular organisms, although the deregulation of cytokines is also characteristic of severe chronic pathologies such as cancer [33]. The histopathology, observed using immunohistochemistry, revealed the levels of inflammatory cytokines including IL-1β (Figure 4a,b) and TNF-α (Figure 4c,d). Our quantification showed that, compared with the control group, the expression of IL-1β and TNF-α was lower in mice treated with 5-FU (by 65.3% and 66.5%), zotarolimus (44.5% and 54.7%), and zotarolimus combined with 5-FU (90.2% and 86.6%) (Figure 4d). However, the activity of IL-1β and TNF-α was 1.7 (*p* < 0.001) and 1.3 (*p* < 0.01) times higher in the zotarolimus group than in the 5-FU group. Moreover, IL-1β and TNF-α expression was lowest in mice treated with zotarolimus combined with 5-FU. This combination treatment resulted in lower expression than that of the mice treated with zotarolimus (by 84.1% for IL-1β and 70.4% for TNF-α) or 5-FU (by 71.8% for IL-1β and 60.1% for TNF-α). Thus, the expression of IL-1β and TNF-α could be largely suppressed in colorectal adenocarcinoma in mice by treatment with a combination of zotarolimus and 5-FU.

**Figure 5:**
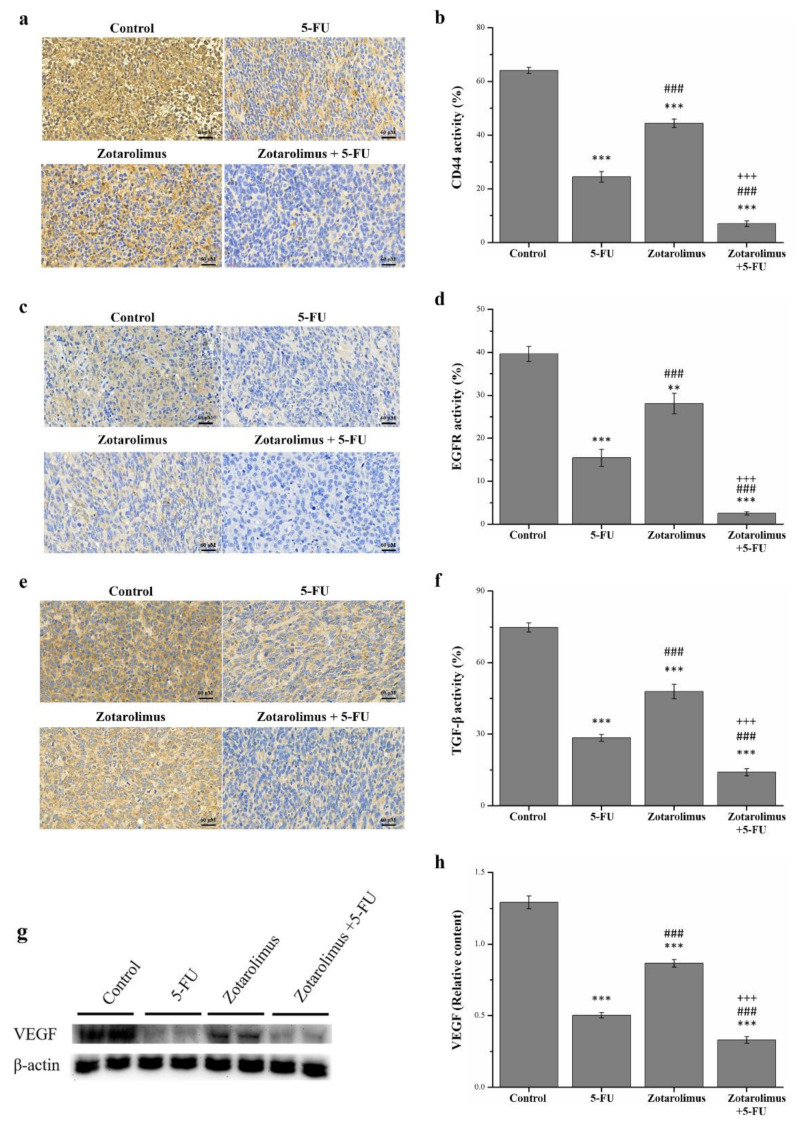
Immunohistochemical expression of (**a**) CD44, (**c**) EGFR, and (**e**) TGF-β, and a comparison of (**b**) CD44, (**d**) EGFR, and (**f**) TGF-β expression levels. Western blot analysis (**g**) showing the expression of metastasis-related factors extracted from tumors in a representative blot. Expression of (**h**) VEGF in the HCT-116 tumor masses from BALB/c nude mice in the control groups and mice administered different treatments, including 5-FU (50 mg/kg/week), zotarolimus (2 mg/kg/day), and a combination of zotarolimus (2 mg/kg/day) and 5-FU (50 mg/kg/week). All data are presented as mean ± standard deviation, *n* = 8 per group. ** *p* < 0.01 and *** *p* < 0.001 compared with the control group. ^###^ *p* < 0.001 compared with the 5-FU-treated group. ^+++^ *p* < 0.001 compared with the zotarolimus-treated group. Scale bars = 60 µm.

Inflammation is also closely associated with cancer, and its reduction or elimination may result in more effective cancer prevention and therapy strategies [34]. Using a Western blot, we determined that the cyclooxygenase-2 (COX-2) expression was lower in the groups treated with 5-FU (*p* < 0.001), zotarolimus (*p* < 0.001), and zotarolimus combined with 5-FU (*p* < 0.001) than in the control group (Figure 5a). Zotarolimus- and 5-FU-treated mice had significantly reduced COX-2 expression compared with the control mice. However, the expression of COX-2 was higher in the zotarolimus-treated mice (*p* < 0.001) than in the 5-FU-treated mice (Figure 5b). The combined treatment of zotarolimus and 5-FU resulted in the lowest COX-2 expression.

### 2.5. Zotarolimus Inhibits Metastasis-Related Factors

The major cause of cancer morbidity and mortality is cancer metastasis, which accounts for approximately 90% of cancer deaths [35]. We selected metastasis-related factors including CD44, EGFR, TGF-β, and VEGF for observations. We used IHC to detect the expression of CD44, EGFR, and TGF-β (Figure 5a,c,d). Compared with the control group, the pathological sections indicated lower CD44, EGFR, and TGF-β expression in the groups treated with 5-FU (CD44: *p* < 0.001; EGFR: *p* < 0.001; TGF-β: *p* < 0.001), zotarolimus (CD44: *p* < 0.001; EGFR: *p* < 0.01; TGF-β: *p* < 0.001), and zotarolimus combined with 5-FU (CD44: *p* < 0.001; EGFR: *p* < 0.001; TGF-β: *p* < 0.001). The quantification results indicated that the levels of CD44 expression in mice treated with 5-FU, zotarolimus, and zotarolimus combined with 5-FU were lower by 61.8%, 30.7%, and 89.0%, respectively, compared with those observed in the control group (Figure 5b). Further, the levels of EGFR expression in the groups treated with 5-FU, zotarolimus, and zotarolimus combined with 5-FU were lower by 61.1%, 29.1%, and 93.7%, respectively (Figure 5d). The levels of TGF-β expression in the groups treated with 5-FU, zotarolimus, and zotarolimus combined with 5-FU were lower by 62.0%, 36.0%, and 81.2%, respectively (Figure 5f). However, the CD44, EGFR, and TGF-β expressions in the zotarolimus group were 1.8, 1.8, and 1.7 times higher, respectively, than those in the 5-FU group. The group treated with zotarolimus combined with 5-FU showed significantly reduced CD44, EGFR, and TGF-β expression compared with those reported for the 5-FU group and zotarolimus group (by 71.3% and 84.2% for CD44, by 83.6% and 91.1% for EGFR, and by 50.7% and 70.7% for TGF-β). The Western blotting revealed similar trends for VEGF (Figure 5g). The expression of VEGF showed a significantly greater decrease in the groups treated with 5-FU (*p* < 0.001), zotarolimus (*p* < 0.001), and zotarolimus combined with 5-FU (*p* < 0.001) when compared with those of the control group (Figure 5h). The VEGF expression in zotarolimus-treated mice (*p* < 0.001) was higher than that in 5-FU-treated mice. Mice treated with zotarolimus combined with 5-FU showed the lowest expression of VEGF. Thus, zotarolimus inhibits metastasis-related factors, and it has the greatest suppressive effect on metastasis when combined with 5-FU.

## 3. Discussion

This study examined the impact of zotarolimus and a combination of zotarolimus and 5-FU on the development of HCT-116 tumors in nude mice. A preliminary experiment showed that zotarolimus (1 mg/kg/day) significantly inhibited the growth of lung adenocarcinoma tumors formed from HCT-116 cells, and zotarolimus combined with 5-FU had a similar inhibiting effect on tumor weight as 5-FU alone (Figure S1). We went on to use 2 mg/kg/day of zotarolimus. The results indicated that zotarolimus inhibited tumor size and weight, and similar effects were observed for the 5-FU-treated mice. However, the inhibitory effect of zotarolimus was not greater than that of 5-FU. Zotarolimus combined with 5-FU showed the highest inhibitory effect when compared with groups treated with 5-FU or zotarolimus alone. As for apoptosis, zotarolimus combined with 5-FU increased the expression of cleaved caspase 3 and ERK phosphorylation and decreased Bcl-2 expression. This indicated that zotarolimus combined with 5-FU could be the most effective in promoting HCT-116 cell apoptosis. From the viewpoint of inflammation, the two groups treated with zotarolimus and zotarolimus combined with 5-FU showed a reduced production of inflammatory factors, including IL-1β, TNF-α, and COX-2. Zotarolimus inhibited metastasis-related factors including CD44, EGFR, TGF-β, and VEGF. Accordingly, the synergistic effect of zotarolimus and 5-FU greatly inhibited the expression of inflammatory and tumor metastasis-related factors.

The mTOR pathway is critical in regulating cell survival, metabolism, growth, and protein synthesis in response to upstream signals under both normal physiological and pathological conditions, particularly in cancer [36]. In addition, the mTOR signaling pathway promotes cell proliferation and metabolism, both of which contribute to tumor initiation and progression [37]. Cancer therapy uses numerous drugs to inhibit the mTOR pathway, such as everolimus, temsirolimus, and ridaforolimus [38,39]. Research conducted using in vivo assays showed that everolimus inhibited breast tumor growth and prolonged the survival of MCF-7-bearing mice [40]. One study suggests that temsirolimus could be useful in treating non-small cell lung cancer (NSCLC) because of its antiproliferative effect and could be a potential treatment for advanced NSCLC, prolonging patient survival [41]. Another study showed that ridaforolimus inhibits mTOR activity, and thus also tumor cell proliferation and VEGF production [42]. In sum, mTOR pathway inhibitors are effective in cancer treatment, and their nature and application have garnered furthered research interest. Our results show that zotarolimus inhibits the growth of HCT-116 cells and could be clinically administered to inhibit the proliferation of colorectal carcinoma cells.

Several studies have shown that combination chemotherapy with an anti-cancer drug and a cell-signal inhibitor achieves a better response rate than the use of one or the other alone. Everolimus, for example, with a high cyclophosphamide dose shows synergistic anti-tumor activity in vivo, as observed in a gastric cancer treatment [43]. Further, we observed that when rapamycin and perifosine mutually suppress the PI3K/Akt/mTOR pathway, they induce synergistic multiple myeloma cell cytotoxicity. This serves as the rationale for clinical trials involving patients with relapsed/refractory multiple myeloma [44]. This study, therefore, investigated the inhibitory and synergistic impact of zotarolimus combined with 5-FU on HCT-116 human CRC cells implanted in mice. Our results revealed that zotarolimus alone had inhibitory effects on HCT-116 cells, and that the synergistic effect of zotarolimus and 5-FU has strong inhibitory effects on tumor growth.

Next, we explored apoptosis. Apoptosis is an important indicator of tumors, and the most effective non-surgical treatment is targeting apoptosis for all types of cancer. This is because apoptosis evasion is a key characteristic of cancer and is nonspecific to the cause or type of the cancer [45]. In our experiments, TUNEL and DAPI revealed the extent of apoptosis in HCT-116 cells in mice treated with zotarolimus alone or a combination of zotarolimus and 5-FU. Zotarolimus alone could enhance the apoptosis of colorectal carcinoma cells, and zotarolimus combined with 5-FU substantially increased the percentage of apoptotic cells. In other words, zotarolimus combined with 5-FU impedes the ability of the cancer cells to evade apoptosis, thus increasing the rate of apoptosis and inhibiting tumor growth. In addition, our Western blots showed higher expression of cleaved caspase 3 and ERK phosphorylation, which are central factors in apoptosis, in mice treated with a combination of zotarolimus and 5-FU. Anti-apoptotic Bcl-2 proteins inhibit apoptosis by blocking the pro-apoptotic Bcl-2 proteins, Bcl-2-associated X protein (BAX), and Bcl-2 homologous antagonist killer (BAK) [46]. Moreover, everolimus induced apoptosis by decreasing Bcl-2, phosphoinositide 3-kinase (PI3K), protein kinase B (AKT), and mTOR expression levels in breast cancer cells [40]. On the other hand, one downstream pathway target of mTOR signaling is S6K, a protein which attaches to mitochondrial membranes and can phosphorylate serine 136 in the pro-apoptotic molecule BAD (Bcl-xL/Bcl-2 associated death promoter) to inactivate it. This phosphorylation interrupts BAD’s binding to the mitochondrial death inhibitors Bcl-XL and Bcl-2 [47]. Zotarolimus also reduces the expression of S6K1 and mTOR (Figure S2). Thus, mTOR can inhibit apoptosis. Accordingly, through the inhibition of the mTOR pathway, the expression of cleaved caspase 3 and ERK phosphorylation showed a significantly greater increase in the groups treated with zotarolimus and zotarolimus combined with 5-FU than in the control group. The apoptosis-related results indicate that the trend in tumor size for all groups is in line with our expectations.

Inflammation is often considered an indicator of cancer development and progression. Chronic inflammation has been observed in the various stages of tumorigenesis, including cellular transformation, survival, proliferation, invasion, angiogenesis, and metastasis [48]. We observed the inflammation factors IL-1β, TNF-α, and COX-2. The cytokines IL-1β and TNF-α are strongly associated with inflammation-driven cancers, including colorectal cancer, and contribute to tumor growth and invasion [49]. Our results indicated that the administration of zotarolimus could reduce the expression of IL-1β and TNF-α levels. In addition, zotarolimus combined with 5-FU significantly reduced inflammation-related factors and increased anti-inflammation factors. IL-6 is a pleiotropic cytokine that critically influences immune responses, inflammation, and haematopoiesis, and is expressed by multiple types of tumor tissue, such as those of breast, prostate, colorectal, and ovarian cancer [50]. IL-10 inhibits tumor growth by hindering several inflammatory and angiogenic factors, including vascular endothelial growth factor, IL-1β, TNF-α, and IL-6 [51]. C-reactive protein (CRP) is a sensitive but non-specific marker of systemic inflammation and is primarily produced by hepatocytes under transcriptional control by IL-6 in response to infection, trauma, surgery, burns, tissue infarction, advanced cancer, and chronic inflammatory conditions [52]. Studies have evaluated the association between circulating CRP levels and colorectal cancer in various populations [53,54]. We found that, compared with the control mice, mice treated with 5-FU, zotarolimus, or zotarolimus combined with 5-FU reported reduced serum IL-6, IL-10, and CRP (Figure S3). The highest levels of serum IL-6, IL-10, and CRP were observed for the group administered with zotarolimus combined with 5-FU, and this was associated with the highest inhibition rate of tumor growth. Thus, zotarolimus can retard tumor growth by attenuating inflammatory cytokine expression and elevating anti-inflammation mediators.

In addition, TNF-α, IL-1β, and other such cytokines are potent inducers of NF-κB [55]. Cancer-causing mutations in the NF-κB pathway can constitutively activate NF-κB, which detrimentally impacts the expression of genes that propagate cell proliferation and prevent apoptosis [56]. NF-κB inhibitor α (IκBα) acts as a negative regulator of the classical NF-κB pathway by maintaining the presence of NF-κB in the cytoplasm [57]. Numerous patients with CRC showing resistance to chemotherapy could account for the constitutive activation of NF-κB [58]. Therefore, NF-κB/IκB-α signaling plays an essential role in various types of cancers. We detected mRNA expression in HCT-116 cells (Figure S4). Zotarolimus and zotarolimus combined with 5-FU increased the expression of IκBα and inhibited NF-κB, and subsequently prevented inflammatory reactions. Thus, zotarolimus could affect the development of tumors by regulating the critical link between inflammation and cancer. In addition, reactive oxygen species (ROS) are a key factor within the inflammatory response. They contribute to CRC progression. ROS are a genotoxic compound that drive the accumulation of mutations within proliferating epithelial cells and contribute to the occurrence and development of malignant tumors [59]. Free radical damage in living organisms can be prevented by antioxidant enzymes, such as superoxide dismutase (SOD) and catalase (CAT) [60]. Their critical mechanism of action creates an integrated system of antioxidant protection related to ROS. Moreover, SOD and CAT reported a significantly greater increase in the group treated with zotarolimus combined with 5-FU than in the other groups (Figure S5). Thus, zotarolimus could reduce oxidative stress in mice with HCT-116 tumors, which highlights the mechanism underlying its anti-tumor effect. 

Another key indicator in oncology research is metastasis. CD44 regulates the phosphoinositide-3-kinase (PI3K)/AKT/mTOR signaling pathway and promotes the migration of cancer cells [61]. The CD44-induced epithelial-mesenchymal transition (EMT), however, is strongly correlated with cancer metastasis and is regulated by TGF-β1. The anti-tumor effect could inhibit the expression of both CD44 and TGF-β [62]. Studies have shown that the proliferation and migration of highly transformed tumor cells is stimulated by TGF-β1, which further causes metastasis and tumor progression [63]. In addition, TGF-β decreases the cytotoxicity of anti-cancer drugs [64]. We observed not only CD44 and TGF-β but also EGFR. EGFR is a major signal transducer of mitogens in cancer pathogenesis and progression upstream of mTOR and is an important target in anti-cancer therapies [65]. Our results showed that zotarolimus and zotarolimus combined with 5-FU greatly reduced the presence of CD44, EGFR, and TGF-β in tumor immunostaining. Therefore, zotarolimus and zotarolimus combined with 5-FU reduced the metastatic ability of the HCT-116 cells. It is likely that reducing the TGF-β expression produced a more prominent effect from the drug therapy, thus inhibiting tumor metastasis in line with the abovementioned reference [64]. As for other metastasis-related factors, the majority of human tumors overexpress VEGF, correlating with the invasiveness, vascular density, metastasis, recurrence, and prognosis [66]. VEGF and EGFR signaling synergize to promote epidermal tumor growth [67]. In our study, zotarolimus combined with 5-FU significantly suppressed tumors by reducing VEGF and EGFR expression. Our results indicate that zotarolimus has an anti-tumor effect and that combining zotarolimus with 5-FU strengthens the effect on suppressing tumor metastasis.

An increasing number of colon cancers are becoming resistant to chemotherapy drugs and forming resistant cell lines. In recent years, the PI3K/AKT/mTOR pathway has emerged as a novel target in efforts to overcome drug resistance [68,69]. Research has shown that in a combinational therapy containing BEZ235, a relatively new inhibitor of the PI3K/Akt/mTOR pathway, and the chemotherapeutic 5-FU, BEZ235 sensitized the HCT-116 colon cancer cells to 5-FU-induced apoptosis [70]. It is important, therefore, to develop combinational therapies using drugs with different mechanisms of action. In our study, the anti-tumor effects of zotarolimus alone on tumor growth were not superior to those of 5-FU in the HCT-116 human CRC cell line. However, zotarolimus combined with 5-FU exhibited excellent inhibition of HCT-116 cell growth in terms of apoptosis, inflammation, and metastasis.

## 4. Materials and Methods

### 4.1. Animal and Cell Lines

For the purpose of this study, six-week-old male BALB/c nude mice were purchased from the National Laboratory Animal Breeding and Research Center, Taipei, Taiwan. Two mice were housed per cage and were provided with sterilized food and water. The mice were maintained at a constant temperature (22 ± 2 °C) and relative humidity (55 ± 5%) with a 12 h/12 h light/dark cycle. All animal use protocols for the experimental mice were reviewed and approved by the Institutional Animal Care and Use Committee (IACUC) of National Chiayi University (IACUC Approval No. 107013). In addition, all procedures adhered to the Guidelines for the Care and Use of Laboratory Animals recommended by Taiwan’s Ministry of Health and Welfare.

An HCT-116 human CRC cell line was purchased from the Bioresource Collection and Research Center (BCRC; Hsinchu, Taiwan). The cells were grown in 90% McCoy’s medium supplemented with 10% fetal bovine serum, 50 IU/ml penicillin, and 50 mg/mL streptomycin (Gibco Laboratories, Grand Island, NY, USA) in a humidified atmosphere of 95% air and 5% CO_2_ at 37 °C. The cells were routinely passaged by removing the medium and overlaying the cell monolayer with 0.25% trypsin and 0.1% EDTA.

### 4.2. Tumor Inoculation and Treatment

The experiment was designed such that the treatment would be initiated when tumor masses were detected, seven days after the HCT-116 cell implantation. After the mice were anesthetized with an intraperitoneal injection of zoletil (Virbac Taiwan, Taipei, Taiwan), they were subcutaneously injected in the posterior leg with a 100 µL cell suspension containing 10^6^ viable HCT-116 cells. On day one of this preliminary experiment, we did not observe any apparent masses in the mice. We re-examined for tumor lesions on day seven. Mice that showed a tumor of diameter 4–5 mm [71] were then divided into four groups. In a subsequent experiment, all mice that had developed apparent tumors by day seven were also randomly divided into four groups (8 mice per group). Group I, the control group, was administered saline on a daily basis via an intraperitoneal injection. Groups II, III, and IV were intraperitoneally injected with 5-FU (50 mg/kg/week) (Sigma; St. Louis, MO, USA), zotarolimus (2 mg/kg/day) (MedChemExpress, Monmouth Junction, NJ, USA), and zotarolimus (2 mg/kg/day) with 5-FU (50 mg/kg/week), respectively. We based the 5-FU dosage on studies showing how 5-FU affects apoptosis, invasion, metastasis, angiogenesis, and growth signal mechanism of colon cancer in mice [72]. The zotarolimus dosage was based on that which is generally administered for lung cancer [30]. We monitored tumor growth every seven days by measuring the greatest and the least diameters using a handheld Peira TM900 imaging device (Peira, Turnhout, Belgium). We calculated tumor volume as follows: V = 0.5 × a × b^2^, where a is the largest diameter and b is the smallest.

### 4.3. Clinical Observations and Histopathological Analysis

We observed the mice on a daily basis for clinical signs and sacrificed them after 28 days. We measured body weight every three days. We collected blood to evaluate hematological parameters under anesthesia at the end of the treatment period. The tumor specimens were divided into two groups. The first group was fixed with 10% formalin and embedded in paraffin. These specimens were then assessed using hematoxylin and eosin staining; the terminal deoxyribonucleotidyl transferse (TdT)-mediated biotin-16-dUTP nick-end labeling (TUNEL assay; APO-BrdU™TUNEL Assay Kit, BDPharmingen, San Diego, CA, USA); and immunohistochemistry (IHC) including IL-1β, TNF-α, CD44, EGFR, and TGF-β. We used IHC staining to assess for IL-1β, TNF-α, CD44, EGFR, and TGF-β in the tumor using primary antibodies against IL-1β, TNF-α, CD44, EGFR, and TGF-β (Merck, Billerica, MA, USA). We measured protein expression through IHC using the TAlink mouse/rabbit polymer detection system from BioTnA (Kaohsiung, Taiwan). We employed Moticam 2300 (Motic Instruments, Richmond, BC, Canada), a high-resolution digital microscope equipped with Motic Images Plus (version 2.0) to capture images and analyze adipocyte size distributions between the control and treated obese mice. The other group was preserved in a freezer at −80 °C and the levels of caspase 3, ERK, Bcl-2, COX-2, and VEGF were examined using Western blotting.

### 4.4. Western Blotting Assay

The mice were euthanized with an overdose of anesthetic combined with carbon dioxide after the experimental period concluded. The tumors of the mice were obtained quickly, coarsely minced, and homogenized. We performed Western blotting, as described elsewhere [18], and used antibodies against β-actin, Bcl-2, COX-2, and VEGF purchased from Sigma-Aldrich Inc. (St. Louis, MO, USA), as well as cleaved caspase 3, phosphorylated ERK (threonine 202/tyrosine 204), and ERK from Cell Signaling Technology (Beverly, MA, USA). Enhanced chemiluminescence reagents (Thermo Scientific, Rockford, MA, USA) were used to produce immunoreactive signals, and UVP ChemStudio (Analytik Jena, Upland, CA, USA) was used to detect these signals. Finally, we quantified protein expression and phosphorylation using ImageJ software from the National Institutes of Health (version 1.8.0, Bethesda, MA, USA).

### 4.5. Statistical Analyses

All results are denoted as mean ± SD. We compared differences between two groups using t-tests. We performed an ANOVA followed by a post-hoc Bonferroni test to determine the differences when analyzing more than two groups. *p*-values less than 0.05, 0.01, and 0.001 were considered significant, very significant, and extremely significant, respectively.

## 5. Conclusions

The study demonstrated a potential synergistic effect of zotarolimus and 5-FU for the treatment of human colon adenocarcinoma. Our findings evidenced that zotarolimus alone inhibits the tumor development of HCT-116 cell in vivo, and combining zotarolimus with a traditional chemotherapy drug such as 5-FU exerts a stronger inhibitory effect. This observation is associated with the inhibition of tumor growth via apoptotic mechanisms in HCT-116 cells, where we observed an elevation of the expression of apoptotic cleaved caspase 3 and ERK phosphorylation and a decrease in the anti-apoptotic expression of Bcl-2. Our study also revealed that both zotarolimus alone and the combination of zotarolimus with 5-FU decreased the production of inflammatory cytokines, including TNF-α, IL-1β, and IL-6, and increased the production of the anti-inflammatory cytokine Il-10 and antioxidant enzyme activities, including those of SOD and CAT. Anti-tumor and anti-inflammation treatments are characterized by the decreased accumulation of COX-2 and NF-κB mRNA and an increase in IκBα mRNA. The inhibition of the mTOR signaling pathway in tumors by zotarolimus reduced CD44, EGFR, TGF-β, and VEGF expression, which could decelerate the migration and invasion of tumor cells in cancer metastasis. Collectively, the in vivo data reveal the pharmacological effects of zotarolimus, which was shown to effectively enhance apoptosis, decrease inflammatory processes, and increase cancer metastasis-suppressing mechanisms. The combination of zotarolimus and 5-FU has a synergistic effect on suppressing tumor growth, although the use of zotarolimus alone has a similar effect. These results may pave the way for providing more options in cancer treatment and the development of new therapeutic strategies for treating CRC.

## Figures and Tables

**Table 1:** Weight of tumors from BALB/c nude mice after sacrifice.

	Tumor Weight (g)	Tumor Inhibition Rate (%)
Control	7.10 ± 1.19	-
5-FU	2.69 ± 0.44 ***	62.1
Zotarolimus	4.51 ± 0.21 ***^###^	36.5
Zotarolimus + 5-FU	1.31 ± 0.15 ***^###+++^	81.5

All data are presented as mean ± standard deviation, *n* = 8. *** *p* < 0.001 compared with control group. ^###^ *p* < 0.001 compared with 5-FU-treated group. ^+++^ *p* < 0.001 compared with the zotarolimus-treated group.

## Data Availability

The data presented in this study are available on request from the corresponding author.

## Supplementary Materials

Figure S1. Tumor weight in BALB/c nude mice post-sacrifice, after mice were administered different treatments including control, 5-FU (50 mg/kg/week), zotarolimus (1 mg/kg/day), and zotarolimus (1 mg/kg/day) combined with 5-FU (50 mg/kg/week). Figure S2. Western blot analysis of (a) mTOR signaling and relative quantitative expression of (b) mTOR phosphorylation and (c) S6K1 phosphorylation in HCT-116 tumors taken from BALB/c nude mice administered different treatments including control, 5-FU (50 mg/kg/week), zotarolimus (2 mg/kg/day), and zotarolimus (2 mg/kg/day) combined with 5-FU (50 mg/kg/week). Figure S3. Changes in serum levels of (a) IL-6, (b) IL-10, and (c) CRP measured in BALB/c nude mice administered different treatments including control, 5-FU (50 mg/kg/week), zotarolimus (2 mg/kg/day), and zotarolimus (2 mg/kg/day) combined with 5-FU (50 mg/kg/week). Figure S4. Relative gene mRNA expression of (a) NF-κB and (b) IκBα using quantitative PCR. All experiments were conducted on the HCT-116 tumor masses of BALB/c nude mice administered different treatments including control, 5-FU (50 mg/kg/week), zotarolimus (2 mg/kg/day), and zotarolimus (2 mg/kg/day) combined with 5-FU (50 mg/kg/week). Figure S5. Changes in serum levels of (a) SOD and (b) CAT measured in BALB/c nude mice administered different treatments including control, 5-FU (50 mg/kg/week), zotarolimus (2 mg/kg/day), and zotarolimus (2 mg/kg/day) combined with 5-FU (50 mg/kg/week). Serum IL-6 and IL-10 levels were measured using commercial kits (ab213749 and ab100697; Abcam, Cambridge, MA, USA). Serum CRP levels were measured using the mouse ELISA kit (SEK50409; Sino Biological, Beijing, China). The Western blotting employed antibody mTOR (Sigma-Aldrich, St. Louis, MO, USA), p-mTOR (Sigma-Aldrich, St. Louis, MO, USA), S6K1 (Cell Signaling, Beverly, MA, USA), and p-S6K1 (Cell Signaling, Beverly, MA, USA). Immunoreactive signals were produced with enhanced chemiluminescence reagent (Thermo Scientific, Rockford, MA, USA) signals. UVP ChemStudio (Analytik Jena, Upland, CA, USA) was then used for signal detection. The quantification of protein expression and phosphorylation was performed using ImageJ (version 1.8.0, NIH, Bethesda, MA, USA). The NF-κB forward, 5′-ATGGCTTCTATGAGGCTGAG-3′, and reverse, 5′-GTTGTTGTTGGTCTGGATGC -3′ [73]; IκB-α forward, 5′- GCCCTTGTCCCTGTCCCTA-3′, and reverse, 5′-GCAGAGTATTTCCCTTTGGTTTGA -3′ [74]. The expression levels for each target gene were calculated relative to the *β-actin* levels and expressed using the 2^−ΔΔCt^ method. Serum SOD and CAT levels were measured using the commercially available colorimetrical kits and used for these procedures (catalase: #K773-100 and SOD: #K335-100; BioVision, Milpitas, CA, USA) following manufacturer-recommended protocols.

## Sample Availability

Not available.
