# Supplementary material for: Anti-Cancer Effects of Zotarolimus Combined with 5-Fluorouracil Treatment in HCT-116 Colorectal Cancer-Bearing BALB/c Nude Mice
Source: Molecules. 2021 Aug 2;26(15):4683. doi: 10.3390/molecules26154683 (PMC8347948; doi:10.3390/molecules26154683)
Supplement: Supplementary file 1 [file molecules-26-04683-s001.zip › molecules-1316970-supplementary.pdf]

molecules

MDPI

Supplementary materials

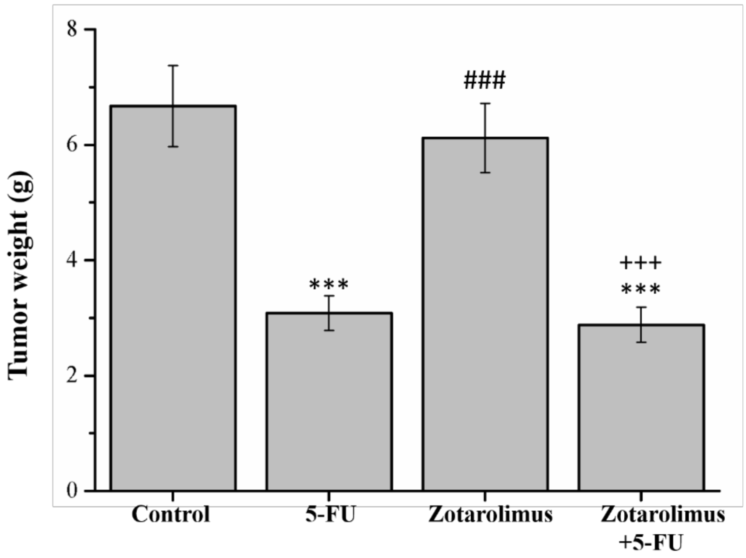

Figure S1. Tumor weight in BALB/cByJNarl mice post-sacrifice, measured after mice were administered different treatments, including control, 5-FU (50 mg/kg/week), zotarolimus (1 mg/kg/day), and zotarolimus (1 mg/kg/day) combined with 5-FU (50 mg/kg/week). All data are mean ± standard deviation, n = 8 per group. $^{***}p < 0.001$ compared with the control group. $^{\#\#\#}p < 0.001$ compared with the 5-FU–treated group. $^{+++}p < 0.001$ compared with the zotarolimus–treated group.

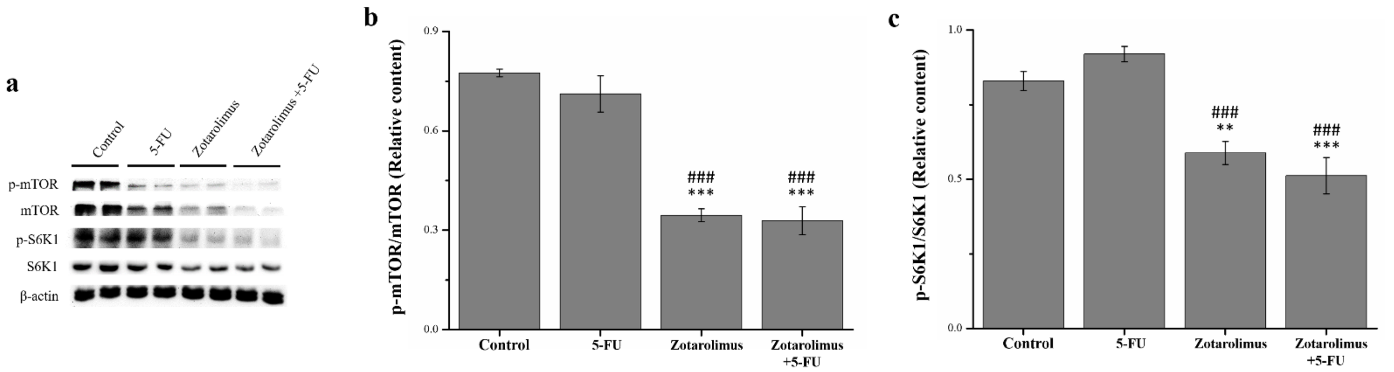

Figure S2. Western blot analysis of (a) mTOR signaling and relative quantitative expression of (b) mTOR phosphorylation and (b) S6K1 phosphorylation in HCT-116 tumors taken from BALB/cByJNarl mice administered different treatments, including control, 5-FU (50 mg/kg/week), zotarolimus (2 mg/kg/day), and a combination of zotarolimus (2 mg/kg/day) and 5-FU (50 mg/kg/week). All data are presented as mean ± standard deviation, n = 8 per group. **p* < 0.05, ***p* < 0.01, and ****p* < 0.001 compared with the control group. ##*p* < 0.01 and ###*p* < 0.001 compared with the 5-FU–treated group. ++*p* < 0.01 compared with the zotarolimus-treated group.

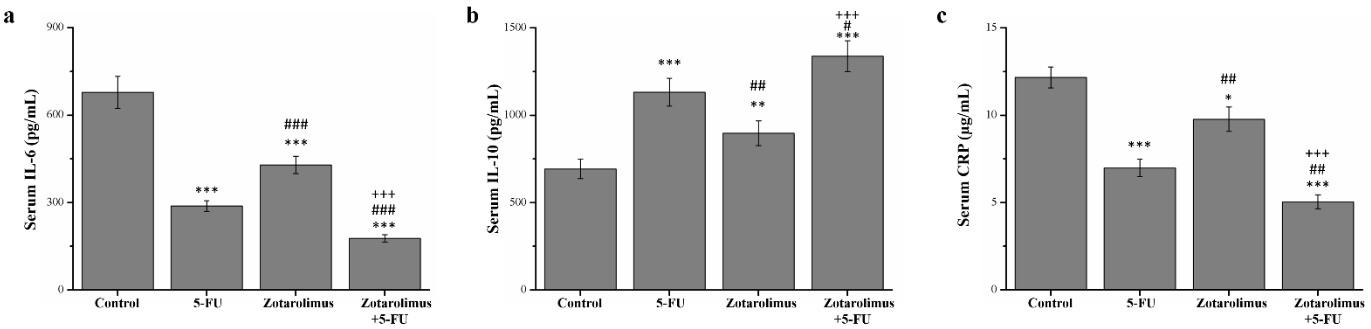

Figure S3. Changes in serum levels of (a) IL-6, (b) IL-10, and (c) CRP measured in BALB/cByJNarl mice administered different treatments, including control, 5-FU (50 mg/kg/week), zotarolimus (2 mg/kg/day), and a combination of zotarolimus (2 mg/kg/day) and 5-FU (50 mg/kg/week). All data are presented as mean ± standard deviation, n = 8 per group. $^{*}p < 0.05$, $^{**}p < 0.01$, and $^{***}p < 0.001$ compared with the control group. $^{\#}p < 0.05$, $^{\#\#}p < 0.01$, and $^{\#\#\#}p < 0.001$ compared with the 5-FU group. $^{+++}p < 0.001$ compared with the zotarolimus group.

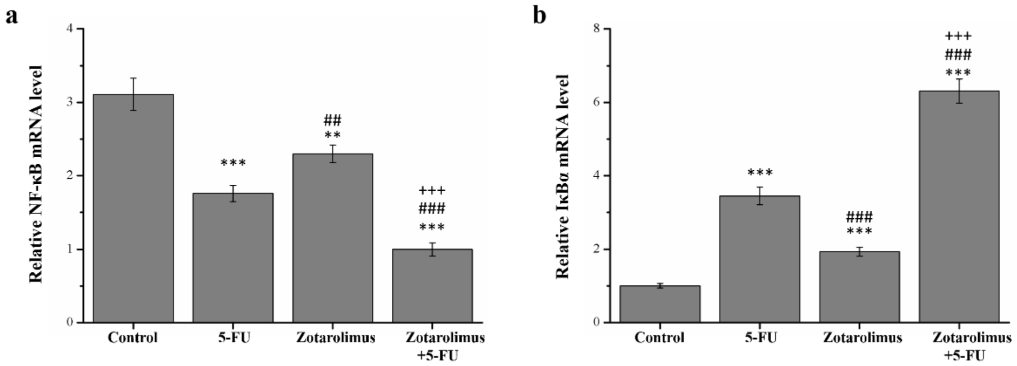

Figure S4. Relative gene mRNA expression of (a) *NF-κB* and (b) *IκBα* using quantitative PCR. All experiments were conducted on the HCT-116 tumor masses from BALB/cByJNarl mice administered different treatments, including control, 5-FU (50 mg/kg/week), zotarolimus (2 mg/kg/day), and a combination of zotarolimus (2 mg/kg/day) and 5-FU (50 mg/kg/week). All data are presented as mean ± standard deviation, n = 8 per group. $^{**}p < 0.01$ and $^{***}p < 0.001$ compared with the control group. $^{\#\#}p < 0.01$ and $^{\#\#\#}p < 0.001$ compared with the 5-FU group. $^{+++}p < 0.001$ compared with the zotarolimus group.

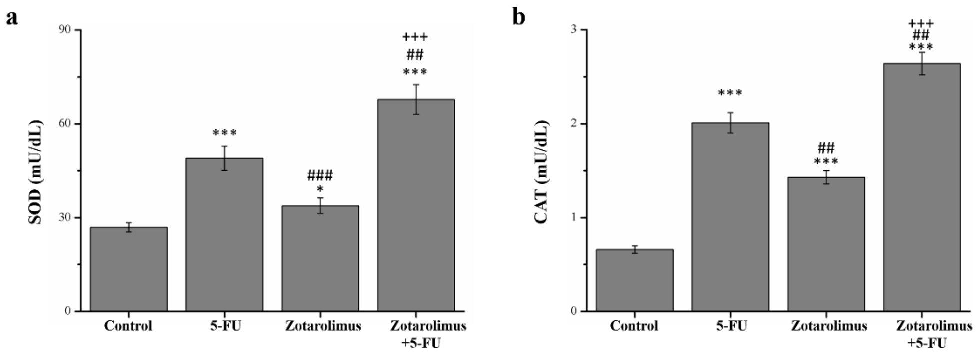

Figure S5. Serum level changes in (a) SOD and (b) CAT measured in BALB/cByJNarl mice administered different treatments, including control, 5-FU (50 mg/kg/week), zotarolimus (2 mg/kg/day), and a combination of zotarolimus (2 mg/kg/day) and 5-FU (50 mg/kg/week). All data are shown as mean ± standard deviation, n = 8 per group. $^{*}p < 0.05$ and $^{***}p < 0.001$ compared with the control group. $^{\#}p < 0.05$, $^{\#\#}p < 0.01$, and $^{\#\#\#}p < 0.001$ compared with 5-FU group. $^{+++}p < 0.001$ compared with zotarolimus group.
